# Small Cell Carcinoma of Gallbladder With Nodal Metastasis Mimicking As Synchronous Malignancy in Gallbladder and Common Bile Duct

**DOI:** 10.7759/cureus.19707

**Published:** 2021-11-18

**Authors:** Naveen Naik, Oseen Shaikh, S. V. Sowmiya, Naveen Kumar Gaur, Gopal Balasubramanian

**Affiliations:** 1 Surgery, Jawaharlal Institute of Postgraduate Medical Education and Research, Puducherry, IND

**Keywords:** neuroendocrine carcinoma, synchronous malignancy, small cell carcinoma, neuroendocrine tumors, gallbladder carcinoma

## Abstract

Small cell carcinoma of the gallbladder is an extremely rare disease. Even with current aggressive and diverse treatments, small cell carcinoma of the gallbladder has an extremely poor prognosis. The presence of synchronous malignancies in the gallbladder and the biliary tract is even rare. Synchronous malignancy can be due to either local spread or metastasis. It can also occur due to field change in the extrahepatic malignancy or can arise de novo as multifocal malignancy. Small cell carcinoma of gallbladder with nodal metastasis mimicking as synchronous malignancy in the gallbladder and distal common bile duct is rare. We report a 74-year-old male who presented with abdominal pain and jaundice. Initial imaging studies were suggestive of the possibility of synchronous malignancy in the gallbladder and common bile duct. However, further imaging studies showed that carcinoma of the gallbladder had metastasis to the lymph nodes, causing external compression to the common bile duct rather than synchronous malignancy. Cytology was diagnostic of small cell carcinoma of the gallbladder. The patient had metastasis to other sites also. The patient underwent endoscopic retrograde cholangiopancreatography with stenting and started on palliative platinum-based chemotherapy with cisplatin and gemcitabine, and he is under regular follow-up.

## Introduction

Gallbladder carcinoma is the most common malignancy of the biliary tree. It is considered the fifth most common malignancy of the world [1]. Neuroendocrine tumors (NETs) of the gallbladder are extremely rare. These specialized cells have traits of both hormone-producing endocrine cells and nerve cells. These tumors can be well-differentiated and poorly differentiated. Small cell carcinoma (SCC) is a poorly differentiated variety with a poor prognosis. Clinically they present similar to adenocarcinoma of the gallbladder, and it is not possible to differentiate clinically. Imaging studies like contrast-enhanced computed tomography (CECT) and magnetic resonance imaging (MRI) help diagnose these tumors. Histopathology is the definitive way of diagnosis. However, preoperative diagnosis is not possible if the patient does not have any distant metastasis, as the patient will be planned for definitive surgery. In the presence of the metastasis, fine-needle aspiration from the gallbladder can clinch the diagnosis, as these patients usually undergo palliative treatment with chemotherapy. Patients with SCC of the gallbladder presenting as metastasis is very common; however, synchronous growth in the gallbladder and common bile duct (CBD) is very rare. However, later imaging suggested metastasis to the lymph nodes compressing the distal CBD in our patient. Hence patient underwent endoscopic retrograde cholangiopancreatography (ERCP) and stenting to reduce jaundice and later started on chemotherapy.

## Case presentation

A 74-year-old man, chronic alcoholic, and smoker presented with a one-month history of progressive jaundice and abdominal pain. He had a history of high-colored urine, clay-colored stools, itching, and reduced appetite, along with significant weight loss in the last few months. On physical examination, he was moderately built and deeply icteric. Abdominal examination revealed a non-tender palpable gallbladder without hepatomegaly. Examination of other systems was within normal limits. Liver function test (LFT) showed increased total bilirubin of 19.85 mg/dL, direct bilirubin of 10.07 mg/dL, and alkaline phosphatase (ALP) of 622 IU/L. Tumor markers like carbohydrate antigen 125 (CA 125) were 45.2 U/mL, carcinoembryonic antigen (CEA) was 5.4 ng/mL, carbohydrate antigen 19-9 (CA 19-9) was more than 1972 U/mL.

**Figure 1:**
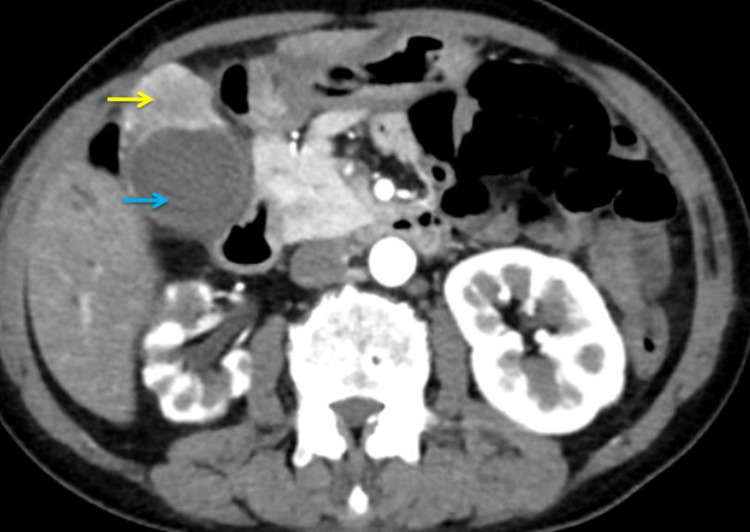
Computed tomography of the abdomen (axial view) showing well-defined enhancing tumor in the gallbladder (yellow arrow), involving segment V of the liver and abdominal wall, with distended gallbladder (blue arrow) without any evidence of cholelithiasis.

Ultrasonography (USG) of the abdomen showed bilobar moderate intrahepatic biliary radical dilatation (IHBRD) and dilated proximal CBD. The CECT abdomen and thorax revealed a soft tissue dense lesion noted at the fundus of the gallbladder measuring 3.2 cm × 3.5 cm × 3.4 cm, with loss of fat planes with segment V of the liver abutting hepatic flexure and infiltrating the abdominal wall musculature (Figure 1).

**Figure 2:**
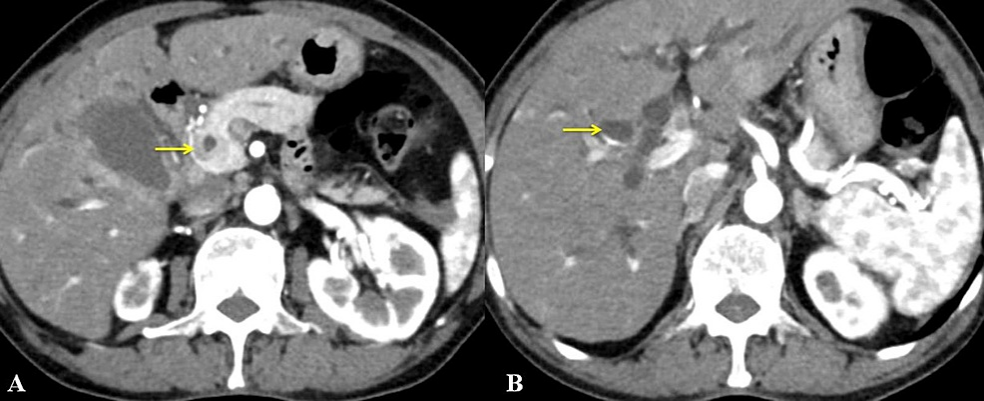
Computed tomography of the abdomen (axial view) showing; (A) well-defined circumferential wall thickening in the distal common bile duct (yellow arrow) and (B) dilated intrahepatic biliary radical (yellow arrow).

An irregular circumferential wall thickening was noted involving the distal CBD with a maximum thickness of 9 mm, causing upstream dilation of CBD and bilobar moderate IHBRD suggestive of synchronous malignancy in the gallbladder and distal CBD. CECT also revealed omental, left adrenal, and thoracolumbar vertebral metastases (Figure 2).

**Figure 3:**
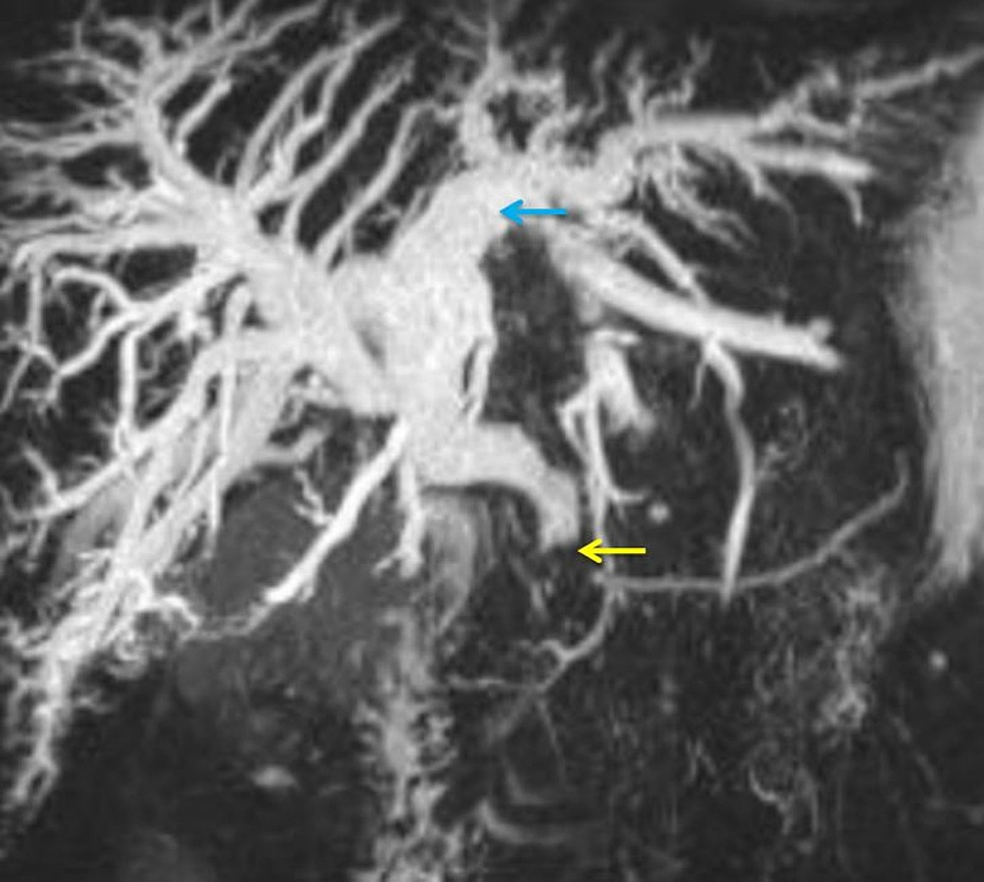
Magnetic resonance imaging (reconstructed T2 image) showing gross dilated intrahepatic biliary radical dilatation (blue arrow) with an abrupt cut-off of the dilated bile duct due to external lymph nodal compression (yellow arrow).

Magnetic resonance cholangiopancreatography (MRCP) and upper abdominal MRI was done, which showed irregular ill-defined lesion noted along the porta abutting the head of the pancreas compressing distal CBD with upstream biliary dilatation measuring about 1.3 cm × 1.2 cm likely to be a metastatic node. Growth at the fundus of the gall bladder shows loss of fat planes with segment V of the liver (Figure 3).

**Figure 4:**
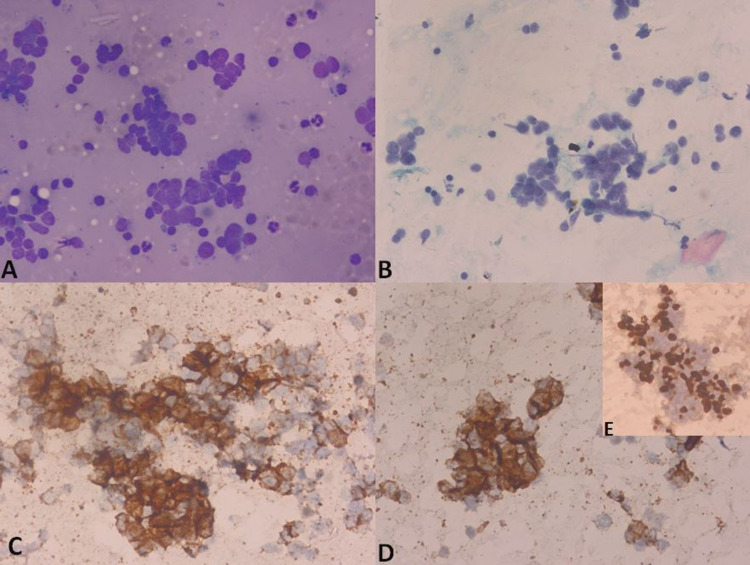
Fine needle aspiration cytology showing; (A) (May Grunwald Giemsa): medium-sized monomorphic tumor cells in loose aggregates and singly scattered, with scanty to absent cytoplasm with nuclear molding, (B) (Papanicolaou stain): pleomorphic tumor cells in loose aggregates with scant to absent cytoplasm, nuclear molding, stippled chromatin, and no significant nucleoli, (C): positive staining for immunocytochemistry for the cluster of differentiation 56 (CD56), (D): positive staining for immunocytochemistry for synaptophysin, and (E) (inset): Ki-67 positivity for more than 50%.

USG guided fine-needle aspiration cytology (FNAC) from the gallbladder growth was done. Cytological examination showed features suggestive of SCC of the gallbladder with moderately cellular and clusters of tumor cells, which are round to oval having scant cytoplasm, high nuclear-cytoplasmic ratio, irregular nuclear membrane, nuclear molding with stippled chromatin in a blood mixed background. Cellblock sections show clusters and singly scattered tumor cells. On immunocytochemistry (IHC), these tumor cells are diffusely positive for cluster of differentiation 56 (CD56) and neuron-specific enolase (NSE); and are negative for synaptophysin and chromogranin. Ki-67 proliferation index was 90% (Figure 4).

As the patient was diagnosed with metastatic SCC of the gallbladder, multidisciplinary tumor board discussion was done, advised for ERCP with stenting to relieve jaundice, itching, and start palliative chemotherapy with cisplatin and gemcitabine. The patient is doing substantially well, and he is under regular follow-up.

## Discussion

Gallbladder carcinoma is the fifth most common malignancy of the gastrointestinal tract. Gallbladder carcinoma is the most common malignancy of the biliary tree. It is considered the fifth most common malignancy of the world [1]. Risk factors for the development of gallbladder malignancy include gallstone disease, choledochal cysts, and chronic inflammation [1]. SCC of the gallbladder is a rare tumor and has an aggressive course.

Primary neuroendocrine tumors (NETs) are a rare heterogeneous group of tumors that can arise anywhere in the body, in the bronchopulmonary system, pancreas, and gastrointestinal system, wherever enterochromaffin cells are. The most common places of NETs are the gastrointestinal system. Occasionally NETs are reported in the hepatobiliary system. The gallbladder is a rare site of occurrence and accounts for less than one percent of the malignancy [2,3].

In 2010, World Health Organization (WHO) classified neuroendocrine neoplasia (NEN) into three types. These include neuroendocrine tumors, neuroendocrine carcinomas (NECs) (small and large cells), and mixed neuroendocrine non-neuroendocrine neoplasm (MiNEN). The term neuroendocrine carcinomas (NECs) differs from neuroendocrine tumors (NETs). NECs have poor differentiation with extensive necrosis, high-cellular atypia, and high-tumor grade. These features of NECs usually correlate with the mitotic count and Ki-67 proliferation index.

NETs are classified as well-differentiated NETs with benign behavior, well-differentiated NETs with uncertain behavior, well-differentiated NECs, and poorly differentiated NECs. The neuroendocrine cells usually produce neuromodulators, neurotransmitters, and neuropeptide hormones. Functionally, NECs can be divided into secretory (functionally active) or non-secretory based on the production of the peptide substances.

Based on the differentiation and grade of the tumor, gallbladder NETs can be classified into four categories. These include well-differentiated NETs (typical carcinoid), well-differentiated NECs (atypical/malignant carcinoid), poorly differentiated NECs (high-grade carcinoma small-cell/large-cell types), and mixed exocrine-endocrine carcinomas. Among these, small- and large-cell carcinomas are high-grade and poorly differentiated. In 2010 WHO established grading of G1 to G3 and staging scheme. Well-differentiated tumors were further divided into G1 (low grade) and G2 (moderate grade), and poorly differentiated NETs were designated as grade G3 [4]. Our patient had SCC of the gallbladder with poor differentiation.

The origin of NETs in the gallbladder is disputable. Usually, the gallbladder wall does not contain neuroendocrine cells. In the literature, many researchers propose that as most NETs accompany cholelithiasis, the inflammations might promote metaplastic changes in the gallbladder epithelium, which leads to the origin of neuroendocrine tumors [5]. So far, the proposed theories for NETs include neuroendocrine metaplasia in patients with cholelithiasis, formation of NETs from gallbladder adenocarcinoma, and transformation of undifferentiated stem cells to neuroendocrine cells [6]. Our patient did not have any cholelithiasis.

Synchronous gallbladder and CBD malignancies are rare. The most common etiological factor associated with synchronous malignancy is an anomalous pancreatic-bile duct junction (APBDJ). Most of these are described in Japanese literature [7]. However, Kurosaki et al. have shown that APBDJ may not be present in all cases of synchronous malignancy of the gallbladder [8]. Our patient did not have an APBDJ anomaly.

The pathway for the development of synchronous malignancies of the biliary tree is not adequate. Shukla et al. have proposed various pathways for developing synchronous malignancies of the biliary tree [9]. Criteria which can differentiate synchronous primaries and metastasis are not clear and not well-defined. Zhai et al. have described certain criteria to distinguish between synchronous primary malignancies and secondary deposits; when simultaneous malignancies of the gallbladder and CBD are discovered. The presence of different histology, lack of anatomical continuity, and typical growth pattern of a primary malignancy are the three criteria proposed to label tumors as synchronous [10]. Synchronous NET of the gallbladder and CBD has never been known. In our case, initially, suspicion of synchronous malignancy was due to circumferential thickening in the distal CBD and proximal dilatation. However, it was clear from the further imaging that it was a metastatic lymph node compressing the distal CBD and causing upstream dilatation.

They are commonly seen in older women and often accompany cholelithiasis.

Clinical symptoms are produced only in patients in whom the peptide substances are not degraded. The most common early manifestation is vague upper abdominal pain. Among non-secretory and secretory NECs, the non-secretory NECs manifest as abdominal pain, weight loss, and jaundice (symptoms of local disease) or symptoms due to metastatic disease. The secretory NECs manifest symptoms related to the secretion of the different peptides and local or metastatic disease symptoms. These can produce various peptides such as serotonin, histamine, prostaglandins, vasoactive intestinal peptide, substance P, and glucagon, thus causing symptoms such as distention, diarrhea, flushing, edema, and wheezing. The usual survival in patients with NETs of the gallbladder is nine months. These tumors commonly metastasize to distant organs such as the liver, lungs, and lymph nodes [11]. Our patient had presented with jaundice and abdominal pain without any functional symptoms.

We cannot solely confirm the diagnosis of NECs preoperatively based on radiological imaging such as USG, CECT scan, MRI scan, and positron emission tomography-computed tomography (PET-CT). Definitive diagnosis can be made only by histopathological examination and IHC staining. In 75% of NECs, the tumor can stain positive for synaptophysin followed by chromogranin A, with a positive rate of 91.9% and 84.8%, respectively [12]. Urinary 5-hydroxyindoleacetic acid (5-HIAA) helps diagnose and monitor the response to the therapy in patients with active secretory NECs. Poorly differentiated NECs appear as atypical, small to intermediate-sized cells, forming ill-defined aggregates with high mitotic figures on histopathological examination. NECs usually have necrosis, angioinvasion, and perineural invasion. Our patient had a poorly differentiated SCC of the gallbladder.

The treatment protocol has not been standardized as it is a rare case, most often seen only in case reports. Not only because of late stages at presentation but also due to aggressiveness of SCC of the gallbladder, it is challenging to establish protocols for its management. Radical surgical resection is the mainstay followed by adjuvant chemotherapy, but whether radical procedures produce better outcomes for advanced stages of small cell carcinomas. In cases of localized pancreaticobiliary SCC, complete surgical resection can have prolonged survival [13]. Locally invasive NECs are usually treated by surgical therapy followed by adjuvant chemotherapy to improve the survival rate. Systemic chemotherapy is the treatment of choice for inoperable or metastasized tumors and in cases where specimen margins contain tumor involvement [14]. The large-cell NECs of the gallbladder have decreased response to chemotherapy, so they have a worse prognosis. Cisplatin or carboplatin and etoposide have been used in the SCC of the gallbladder. The chemotherapeutic agents such as topotecan, irinotecan, taxanes, and gemcitabine or gemcitabine with platinum agents have been reserved for salvage with variable success [15]. Streptozocin with fluorouracil or doxorubicin was used for pancreatic NETs. Recently, a few new drugs have been under assessment for gastroenteropancreatic NETs, which target angiogenesis and intracellular signal pathways [16].

Patients of SCC having a metastatic bone disease or spinal cord compression can be benefited from local radiation therapy. Modalities such as radiofrequency ablation, transarterial chemoembolization, and transarterial-radio ablation can be used in liver metastasis patients. Duffy et al. reported that the median survival of gallbladder NEC was less than ten months, lower than the median survival of gallbladder carcinoma [17,18]. Overall, the poor prognostic factors include high mitotic rate, high Ki-67 index, poorly differentiated-type neuroendocrine carcinomas, and tumor invasion to the local structures. Our patient was started on cisplatin and gemcitabine-based chemotherapy.

## Conclusions

NECs of the gallbladder with nodal metastasis mimicking as synchronous malignancy in the gallbladder and distal CBD are rare. SCC of the gallbladder has an extremely low incidence and poor prognosis because of its late-stage presentation and aggressiveness. Early diagnosis and aggressive treatment, including resection and chemoradiation, have produced good clinical outcomes. Unfortunately, in our case, the SCC of the gallbladder was metastatic, and hence the patient was planned for palliative treatment.
